# Differences in TCDD-elicited gene expression profiles in human HepG2, mouse Hepa1c1c7 and rat H4IIE hepatoma cells

**DOI:** 10.1186/1471-2164-12-193

**Published:** 2011-04-15

**Authors:** Edward Dere, Andrea W Lee, Lyle D Burgoon, Timothy R Zacharewski

**Affiliations:** 1 Department of Biochemistry & Molecular Biology, Michigan State University, East Lansing, Michigan, 48824, USA; 2 Center for Integrative Toxicology, Michigan State University, East Lansing, MI 48824, USA

## Abstract

**Background:**

2,3,7,8-Tetrachlorodibenzo-*p*-dioxin (TCDD) is an environmental contaminant that elicits a broad spectrum of toxic effects in a species-specific manner. Current risk assessment practices routinely extrapolate results from *in vivo *and *in vitro *rodent models to assess human risk. In order to further investigate the species-specific responses elicited by TCDD, temporal gene expression responses in human HepG2, mouse Hepa1c1c7 and rat H4IIE cells were compared.

**Results:**

Microarray analysis identified a core set of conserved gene expression responses across species consistent with the role of AhR in mediating adaptive metabolic responses. However, significant species-specific as well as species-divergent responses were identified. Computational analysis of the regulatory regions of species-specific and -divergent responses suggests that dioxin response elements (DREs) are involved. These results are consistent with *in vivo *rat vs. mouse species-specific differential gene expression, and more comprehensive comparative DRE searches.

**Conclusions:**

Comparative analysis of human HepG2, mouse Hepa1c1c7 and rat H4IIE TCDD-elicited gene expression responses is consistent with *in vivo *rat-mouse comparative gene expression studies, and more comprehensive comparative DRE searches, suggesting that AhR-mediated gene expression is species-specific.

## Background

2,3,7,8-Tetrachlorodibenzo-*p*-dioxin (TCDD) is a ubiquitous environmental contaminant that elicits a broad spectrum of biochemical and physiological effects in a species-specific manner [1]. These effects include lethality, cancer, developmental abnormalities, immunotoxicity, skin lesions, hepatotoxicity, and xenobiotic enzyme metabolism induction. They result from altered gene expression mediated by the aryl hydrocarbon receptor (AhR), a ligand activated transcription factor [1,2]. Briefly, TCDD binds to the cytoplasmic AhR causing nuclear translocation and heterodimerization with the AhR nuclear translocator (ARNT). The heterodimer then binds to specific DNA elements, termed dioxin response elements (DREs), within the regulatory regions of targeted genes to modulate expression, resulting in downstream physiological responses [3]. Although the structure and function of the AhR are highly conserved [4], the sensitivity to and the responses elicited by TCDD vary widely across species, suggesting TCDD and related compounds may activate species-specific AhR-mediated gene expression networks.

Risk assessment assumes that there is a conserved mode of action and comparable toxic responses between species. However, there are inherent differences between species that compromise the extrapolation of rodent data to estimate potential human risks. Moreover, there is a discord between preclinical animal testing compared to human clinical trials regarding toxicity [5]. Similarly, species differ widely in response to TCDD exposure. For example, LD_50 _values range from 1 μg/kg in the guinea pig [6] to >1000 μg/kg for the hamster, and several responses exhibit species-specific sensitivities and toxicities. These differential effects are not attributed to differences in binding affinity or AhR complex stability [7-9]. Collectively, these data suggest that although the AhR is well conserved, subsequent differential gene expression responses are species-specific.

To further investigate differences in TCDD elicited differential gene expression, global gene expression was assessed in human HepG2, mouse Hepa1c1c7 and rat H4IIE hepatoma cells following treatment with TCDD. Comparative analysis indicates there are significant differences in gene expression between species, suggesting AhR-mediated gene expression may not be conserved.

## Results

### Temporally Conserved Gene Expression Responses Elicited by TCDD

**Figure 1:**
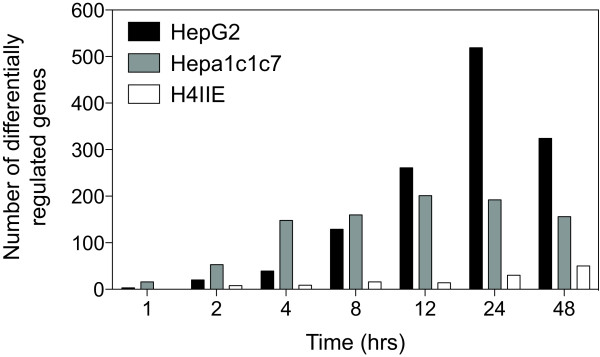
**Number of TCDD-elicited differentially expressed genes in human HepG2, mouse Hepa1c1c7 and rat H4IIE**. Global gene expression changes were detected using cDNA microarray analysis of cells treated with 10 nM TCDD for 1, 2, 4, 8, 12, 24 and 48 hrs. Differentially expressed genes are defined as having P1(t) > 0.999 and |fold change| > 1.4-fold at one or more time points.

**Figure 2:**
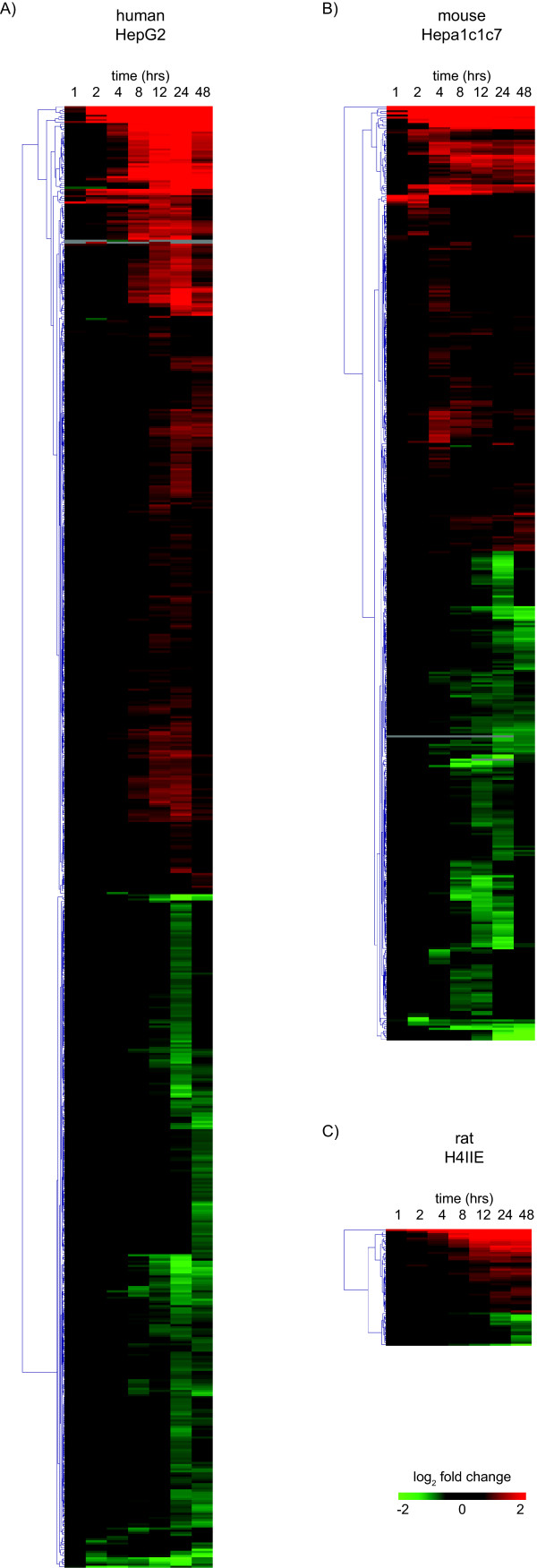
**Hierarchical clustering of differentially expressed genes (P1(t) > 0.999 and |fold change| > 1.4) from cDNA microarray time course studies in HepG2, Hepa1c1c7 and H4IIE hepatoma cells**.

Species-specific, cDNA microarrays were used to profile the temporal gene expression elicited by TCDD in human HepG2, mouse Hepa1c1c7 and rat H4IIE cells. The microarrays queried 6995, 8478 and 5169 unique human, mouse and rat genes, respectively (Table 1). Empirical Bayes analysis identified 691, 439 and 57 differentially expressed genes (P1(t) > 0.999 and |fold change| > 1.4) in HepG2, Hepa1c1c7 and H4IIE cells, respectively. Complete cDNA microarray data sets are provided in Additional files 1, 2, 3. HepG2 cells were the most responsive as indicated by both the overall number of differentially expressed genes as well as the number of responsive genes at each time point (Figure 1). H4IIE cells exhibited significantly less differentially expressed genes, partially explained by the smaller microarray and the immaturity of rat genome annotation compared to the human and mouse. Differentially expressed genes were hierarchically clustered based on Euclidian distance and distinct clusters of temporal gene expression patterns were identified (Figure 2).

**Table 1:** Gene coverage of species-specific cDNA microarray platforms and number of differentially regulated genes

	Human HepG2		Mouse Hepa1c1c7		Rat H4IIE	
			
	Total	Differentially Regulated^a^	Total	Differentially Regulated^a^	Total	Differentially Regulated^a^
Unique Genes^b^	6,995	624	8,478	438	5,169	56
Orthologs^c^	6,825	616	8,233	432	4,871	52

^a^|fold change| > 1.4 and P1(t) > 0.999

^b^based on Entrez GeneID

^c^based on HomoloGeneID

**Figure 3:**
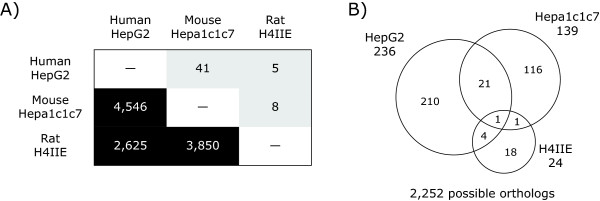
**Cross-species comparison of TCDD-elicited temporal gene expression responses using cDNA microarrays**. (A) The lower half (dark boxes) of the matrix show the number of orthologs represented between pairs of species-specific cDNA microarray platforms. The upper half (grey boxes) of the matrix provides the number of TCDD elicited differentially expressed orthologs between pairs of cell lines. (B) The Venn diagram shows the overlap of all differentially expressed genes (P1(t) > 0.999 and |fold change| > 1.4) from 2,252 possible orthologs represent across all three microarray platforms. Additional files 4, 5, 6 lists the shared responses between pairs of species.

**Figure 4:**
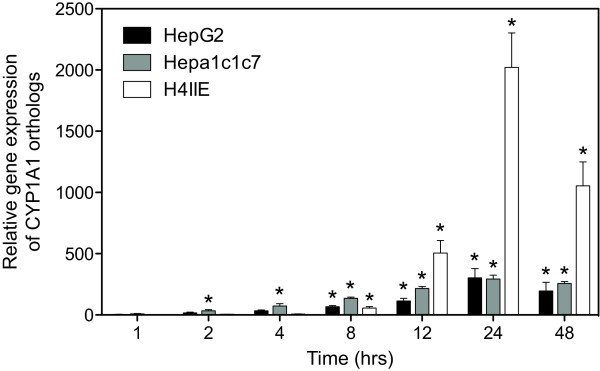
**QRTPCR verification of the conserved induction of CYP1A1 across the human HepG2, mouse Hepa1c1c7 and rat H4IIE cell lines**. The same RNA used for microarray analysis was examined by QRTPCR. Fold changes were calculated relative to time-matched vehicle controls. Error bars represent the standard error of measurement for the average fold change and the asterisk (*) indicates p < 0.05.

Pair-wise comparisons of differentially expressed genes were conducted using HomoloGene (build 35) defined orthologs (Figure 3A). Human and mouse cDNA microarrays shared 4546 orthologous genes, however only 0.9% (41 orthologs; Additional file 4) were differentially regulated by TCDD in HepG2 and Hepa1c1c7 cells. Comparison of the rodent platforms identified 3850 orthologs with only 0.2% (8 orthologs; Additional file 5) responding in both Hepa1c1c7 and H4IIE cells. The lack of conserved ortholog differential expression in Hepa1c1c7 and H4IIE cells is consistent with the reported differences in differential expression observed *in vivo *between mice and rats [10-12]. Time dependent profiling of HepG2 and H4IIE gene expression identified only 5 conserved responses out of 2625 possible orthologs, representing only 0.2% (Additional file 6). Comparative analysis across all three species with 2252 shared orthologous probes identified only one ortholog that was differentially regulated in all three cell lines (immediate early response 3, IER3; HomoloGene ID 2894). Note that other members of the AhR gene battery, namely CYP1A1, ALDH3A1 and NQO1, were not present across all of the cDNA microarray platforms. However, their responses were also conserved across all three cell lines when measured using QRTPCR. The results for CYP1A1 are shown in Figure 4.

### Identification of Putative Primary Gene Expression Responses

In order to further investigate AhR-mediated responses, TCDD-elicited differential gene expression was examined in the presence of cycloheximide (CHX), a protein translation inhibitor. Putative primary responses were defined in this study as those where CHX co-treatment either maintained or enhanced the response elicited by TCDD, while responses that were attenuated or blocked by CHX co-treatment were classified as secondary responses based on their assumed dependence on additional protein translation. cDNA microarray analysis confirmed the superinduction of CYP1A1 mRNA in CHX co-treated Hepa1c1c7 cells [13,14], consistent with the superinduction in human MCF10A cells treated with TCDD [15]. Additionally, Hepa1c1c7 ARNT-deficient c4 mutants treated with TCDD did not exhibit induction of the prototypical AhR battery genes including CYP1A1 [16]. Collectively, these results indicate that CYP1A1 is a primary gene expression response consistent with the direct interaction of the AhR with DREs within the promoter region.

For each species, differentially expressed orthologs were classified as primary or secondary responses based on CHX co-treatment studies at both 4 and 12 hrs. Overall 61, 38 and 2 human, mouse and rat orthologs, respectively, were considered primary responses (Additional files 7, 8, 9). Furthermore, 45, 12 and 10 orthologous genes in the HepG2, Hepa1c1c7 and H4IIE cells were classified as secondary AhR responses (Additional files 10, 11, 12). Comparative examination of the CHX co-treatment data suggested that each cell line had its own unique set of primary responsive orthologs.

### Whole-Genome Analysis of Conserved TCDD-Elicited Gene Expression Responses

The lack of whole genome coverage on the human, mouse and rat cDNA microarrays limited the number of orthologs that could be investigated. Therefore, whole genome expression analysis was performed at 24 hrs, one of the most active time points in terms of the number of differentially expressed genes (Figure 1), using 4 × 44 k Agilent oligonucleotide microarrays. Each microarray contained more than 41,000 individual probes, representing more than 18,000 unique genes (Table 2). Despite the increased coverage, the number of TCDD elicited differentially expressed genes were surprisingly small relative to the cDNA microarray results. For example, the human Agilent microarray consisted of 19,406 known genes, representing a 2.8-fold increase in coverage compared to the human cDNA microarray. However, only 899 unique genes were differentially expressed, a modest increase from the 691 genes identified using cDNA microarrays. Similarly, only 519 and 121 genes were responsive in the Hepa1c1c7 and H4IIE cells, respectively. Complete Agilent microarray data sets are provided in Additional files 13, 14, 15.

**Table 2:** Gene coverage of species-specific Agilent microarray platforms and number of differentially regulated genes

	Human HepG2		Mouse Hepa1c1c7		Rat H4IIE	
			
	Total	Differentially Regulated^a^	Total	Differentially Regulated^a^	Total	Differentially Regulated^a^
Unique Genes^b^	18,499	865	20,929	508	18,244	129
Orthologs^c^	16,781	828	17,543	477	15,705	116

^a^|fold change| > 1.4 and P1(t) > 0.999

^b^based on Entrez GeneID

^c^based on HomoloGeneID

**Figure 5:**
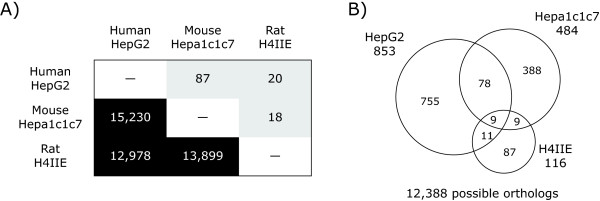
**Cross-species comparison of TCDD-elicited gene expression responses at 24 hrs using 4 × 44 k Agilent microarrays**. (A) The lower half (dark boxes) of the matrix show the number or orthologous genes represented between pairs of species-specific microarrays. The upper half (grey boxes) provides the number of TCDD-elicited differentially expressed orthologs between pairs of cell lines. (B) The 3-way Venn diagram shows the overlap of all differentially expressed genes (P1(t) > 0.999 and |fold change| > 1.4) from 12,388 possible orthologous genes represented across all three platforms. The 9 genes differentially expressed in all three cell lines are listed in Table 3. Additional files 16, 17, 18 lists the shared responses between pairs of species.

The use of whole genome microarrays also increased the number of orthologs that could be examined (Figure 5A). As seen with the cDNA microarray dataset pair-wise comparisons, HepG2 and Hepa1c1c7 cells shared the greatest number of TCDD responsive orthologs (Additional files 16, 17, 18). Ortholog coverage between all three species increased from 2252 on the cDNA microarrays to 12,388 across the Agilent platforms. Comparative analysis (P1(t) > 0.999 and |fold change| > 1.4) identified only 10 orthologs that were differentially expressed by TCDD at 24 hrs (Figure 5B; Table 3). Whole genome expression profiling identified the species-conserved induction of CYP1A1, TIPARP and UGT1A6 in all three cell lines. Despite this increased coverage, the number of differentially expressed orthologs across all three cell lines remained small, consistent with our cDNA microarray results.

**Table 3:** List of common genes identified as differentially expressed by TCDD treatment from whole genome Agilent microarray analysis.

Gene Symbol	Gene Name	Homologene ID	Fold change^a^					
			
			Human		Mouse		Rat	
NQO1	NAD(P)H dehydrogenase, quinone 1	695	3.22	▲	4.88	▲	4.82	▲
CCND1	cyclin D1	1334	1.57	▲	-1.56	▼	-1.47	▼
ID3	inhibitor of DNA binding 3, dominant negative helix-loop-helix protein	1633	1.74	▲	-1.83	▼	-1.68	▼
TIPARP	TCDD-inducible poly(ADP-ribose) polymerase	9167	1.47	▲	7.05	▲	2.91	▲
POC1A	POC1 centriolar protein homolog A	51460	-1.64	▼	-1.67	▼	-1.57	▼
CYP1A1	cytochrome P450, family 1, subfamily A, polypeptide 1	68062	13.64	▲	153.52	▲	134.16	▲
GSTA5	glutathione S-transferase A5	74378	-2.47	▼	1.47	▲	5.85	▲
UGT1A6	UDP glucuronosyltransferase 1 family, polypeptide A6	85959	2.09	▲	2.24	▲	2.06	▲
MT1E	metallothionein 1E	108228	4.89	▲	-1.66	▼	2.65	▲

*^a^*Maximum absolute foldchange determined by microarray analysis

*^b^*Differentially regulated genes with P1(t) > 0.999 and |foldchange| > 1.4

### Species-Specific & Species-Divergent Gene Expression Responses

**Figure 6:**
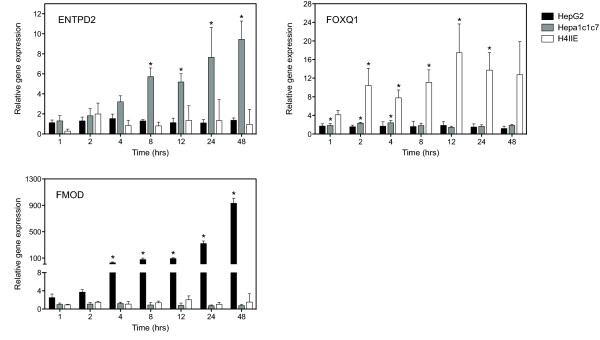
**QRTPCR verification of examples of species-specific orthologous gene expression responses identified from whole-genome microarray analysis at 24 hrs in the human HepG2, mouse Hepa1c1c7 and rat H4IIE cell lines**. The same RNA used for microarray analysis was examined by QRTPCR. Representative orthologs that exhibited significant expression in only one species were verified; FMOD, ENTPD2 and FOXQ1 and were differentially expressed in only the HepG2, Hepa1c1c7 and H4IIE cells respectively. Fold changes were calculated relative to time-matched vehicle controls. Error bars represent the standard error of measurement for the average fold change and the asterisk (*) indicates p < 0.05.

Whole genome comparative analysis of HepG2, Hepa1c1c7 and H4IIE responses identified genes that were species-specific, i.e. differentially expressed in only a single species. For example, microarray analysis at 24 hrs found that fibromodulin (FMOD, HomoloGene ID 1530) was significantly up-regulated 17.2-fold in the HepG2 cells while no significant change in expression was detected in the Hepa1c1c7 and H4IIE cells. Other examples of mouse and rat specific responses include forkhead box Q1 (FOXQ1, HomoloGene ID 7359) and ectonucleoside triphosphate diphosphohydrolase 2 (ENTPD2, HomoloGene ID 20333). FOXQ1 was up-regulated 5.9-fold in the Hepa1c1c7 cells while ENTPD2 was up-regulated 3.2-fold in the H4IIE cells. For both of these genes, the corresponding ortholog in the other two species did not exhibit significant differential expression. The species-specific responses of FMOD, FOXQ1 and ENTPD were verified using QRTPCR (Figure 6). These responses are consistent with previous reports of species-specific TCDD elicited hepatic gene expression characterized in mice and rats [10,11].

**Figure 7:**
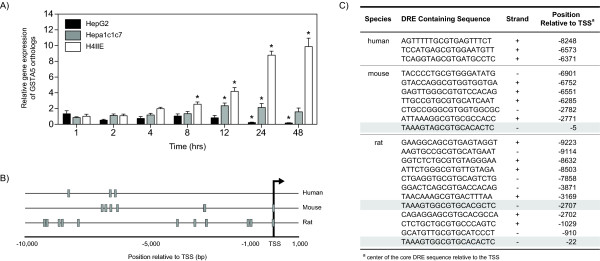
**Comparative analysis of GSTA5 orthologs**. (A) QRTPCR verification of the divergent expression of GSTA5 orthologs in human HepG2, mouse Hepa1c1c7 and rat H4IIE cell lines. Fold changes were calculated relative to time-matched vehicle controls. Error bars represent the standard error of measurement for the average fold change and the asterisk (*) indicates p < 0.05. (B) Gene regulatory regions (-10,000 to 1,000 bp relative to the transcription start site [TSS]) were computationally searched for the 5 bp DRE core (GCGTC) and indicated by the grey boxes. Each core was extended by the flanking 7 bp, and the resulting 19 bp sequence (C) was compared to a consensus DRE sequence using a position weight matrix developed from *bona fide *functional DREs. The shaded rows in (C) indicate the highly similar mouse and rat DRE sequences (Euclidean ≤ 3.0).

Comparative analysis of the orthologous gene expression responses in HepG2, Hepa1c1c7 and H4IIE datasets identified 10 orthologs that were differentially expressed by TCDD across the three models. Further analysis indicated that not all of these responses were directionally conserved, i.e. pattern of expression was not the same in all species. For example, IER3 was induced 1.4-fold in Hepa1c1c7 cells at 4 hrs and 1.5-fold in H4IIE cells at 12 hrs, but repressed -1.9-fold in HepG2 cells at 24 hrs. Likewise, glutathione S-transferase alpha 5 (GSTA5, HomoloGene ID 74378) was induced 1.5-fold in Hepa1c1c7 cells and 4.9-fold in H4IIE cells, but down-regulated 2.5-fold in HepG2 cells. QRTPCR confirmed the divergent expression of GSTA5 in the rat and human cell lines, but found Hepa1c1c7 cells were relatively non-responsive (Figure 7A). Two other orthologs, cyclin D1 (CCND, HomoloGene ID 1334) and inhibitor of DNA binding 3 (ID3, HomoloGene ID 1633), also exhibited divergent expression between species, where the orthologs were down-regulated in the rodent cell lines but induced in HepG2 cells (Table 3). Species-specific and species-divergent responses have also been reported *in vivo *for TCDD elicited hepatic differential gene expression in mice and rats [10,11].

### Functional Enrichment and Network Analysis

**Figure 8:**
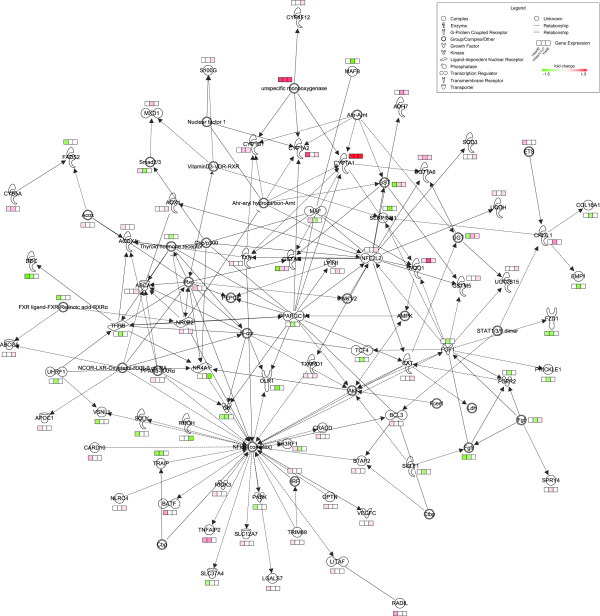
**Gene interaction network of TCDD-responsive orthologs functionally related to lipid metabolism**. Molecules are represented by nodes connected by arrows to indicate either direct or indirect relationships (dotted lines). Colors in the gene expression boxes represent the direction and magnitude of the gene expression responses from the Agilent microarrays. The network visualizes species-conserved and -divergent gene expression responses elicited by TCDD in the human HepG2, mouse Hepa1c1c7 and rat H4IIE cell lines.

Microarray analysis identified a small subset of TCDD responsive genes in the entire genome of each species. Differentially expressed genes elicited by TCDD in each cell line were clustered for functional enrichment based on GO terms (enrichment score ≥ 1.5). Enriched HepG2, Hepa1c1c7, and H4IIE responses clusters included GO terms related to xenobiotic exposure (GO:0009410) and metabolism (GO:0006805) (Tables 4, 5, 6), indicating that these processes are conserved in all three cell lines, and consistent with reported *in vivo *studies using rats and mice[10-12]. However, other enriched GO terms, such as those related to lipid metabolism and transport, were only enriched in HepG2 and H4IIE cell lines. Genes functionally related to lipid metabolism were further analyzed using Ingenuity Pathway Analysis to identify a network of TCDD-responsive orthologs, and species-conserved and -specific responses (Figure 8). Although GO terms related to lipid metabolism were enriched HepG2 and H4IIE differential gene expression data sets, there were few orthologs that exhibited differential regulation in both cell lines based on the fold-change and statistical cutoffs used. *In vivo *rat studies also identified enrichment of genes related to lipid metabolism following TCDD treatment, suggesting that H4IIE cells may be predictive of TCDD-induced perturbations of this pathway rats.

**Table 4:** Functional enrichment analysis of differentially regulated^a ^genes elicited by TCDD in HepG2 cells using DAVID.

Category	Term	Gene count	Fold enrichment	P-value
**Enrichment Score: 2.99**				
GOTERM_BP_3	GO:0009410 ~ response to xenobiotic stimulus	10	8.03	1.54E-06
GOTERM_BP_3	GO:0006805 ~ xenobiotic metabolic process	9	8.32	4.92E-06
GOTERM_BP_3	GO:0017144 ~ drug metabolic process	3	3.46	2.13E-01
GOTERM_BP_3	GO:0006800 ~ oxygen and reactive oxygen species metabolic process	4	1.12	7.00E-01
				
**Enrichment Score: 2.71**				
GOTERM_BP_3	GO:0022403 ~ cell cycle phase	40	1.80	4.36E-04
GOTERM_BP_3	GO:0048285 ~ organelle fission	25	2.03	1.35E-03
GOTERM_BP_3	GO:0000278 ~ mitotic cell cycle	34	1.70	3.07E-03
GOTERM_BP_3	GO:0022402 ~ cell cycle process	45	1.48	8.10E-03
				
**Enrichment Score: 2.30**				
GOTERM_BP_3	GO:0006629 ~ lipid metabolic process	70	1.65	3.82E-05
GOTERM_BP_3	GO:0008610 ~ lipid biosynthetic process	25	1.47	5.48E-02
GOTERM_BP_3	GO:0044255 ~ cellular lipid metabolic process	37	1.34	6.10E-02
				
**Enrichment Score: 2.03**				
GOTERM_BP_3	GO:0006790 ~ sulfur metabolic process	15	2.41	3.58E-03
GOTERM_BP_3	GO:0006518 ~ peptide metabolic process	9	3.20	6.37E-03
GOTERM_BP_3	GO:0051186 ~ cofactor metabolic process	20	1.91	8.81E-03
GOTERM_BP_3	GO:0006519 ~ cellular amino acid and derivative metabolic process	28	1.48	3.94E-02
				
**Enrichment Score: 1.63**				
GOTERM_BP_3	GO:0005975 ~ carbohydrate metabolic process	43	1.55	4.51E-03
GOTERM_BP_3	GO:0044262 ~ cellular carbohydrate metabolic process	32	1.56	1.40E-02
GOTERM_BP_3	GO:0016051 ~ carbohydrate biosynthetic process	12	2.09	2.79E-02
GOTERM_BP_3	GO:0005996 ~ monosaccharide metabolic process	19	1.62	4.67E-02
GOTERM_BP_3	GO:0046165 ~ alcohol biosynthetic process	6	2.52	8.71E-02
				
**Enrichment Score: 1.57**				
GOTERM_BP_3	GO:0010817 ~ regulation of hormone levels	17	2.11	6.67E-03
GOTERM_BP_3	GO:0034754 ~ cellular hormone metabolic process	9	2.87	1.22E-02
GOTERM_BP_3	GO:0042446 ~ hormone biosynthetic process	4	2.38	2.34E-01

*^a^*Differentially regulated genes with P1(t) > 0.999 and |foldchange| > 1.4

**Table 5:** Functional enrichment analysis of differentially regulated^a ^genes elicited by TCDD in Hepa1c1c7 cells using DAVID.

Category	Term	Gene count	Fold enrichment	P-value
**Enrichment Score: 18.67**				
GOTERM_BP_3	GO:0000278 ~ mitotic cell cycle	43	5.95	7.67E-21
GOTERM_BP_3	GO:0022402 ~ cell cycle process	52	4.50	1.15E-19
GOTERM_BP_3	GO:0022403 ~ cell cycle phase	47	4.87	3.67E-19
GOTERM_BP_3	GO:0048285 ~ organelle fission	36	6.14	6.31E-18
				
**Enrichment Score: 2.05**				
GOTERM_BP_3	GO:0006805 ~ xenobiotic metabolic process	4	11.20	4.67E-03
GOTERM_BP_3	GO:0009410 ~ response to xenobiotic stimulus	4	8.96	9.05E-03
GOTERM_BP_3	GO:0006725 ~ cellular aromatic compound metabolic process	9	2.75	1.66E-02
				
**Enrichment Score: 2.04**				
GOTERM_BP_3	GO:0048522 ~ positive regulation of cellular process	58	1.51	1.41E-03
GOTERM_BP_3	GO:0042127 ~ regulation of cell proliferation	27	1.72	7.62E-03
GOTERM_BP_3	GO:0008284 ~ positive regulation of cell proliferation	14	1.69	7.12E-02
				
**Enrichment Score: 1.62**				
GOTERM_BP_3	GO:0051640 ~ organelle localization	7	4.36	5.10E-03
GOTERM_BP_3	GO:0051651 ~ maintenance of location in cell	4	5.38	3.68E-02
GOTERM_BP_3	GO:0051235 ~ maintenance of location	4	4.07	7.35E-02

*^a^*Differentially regulated genes with P1(t) > 0.999 and |foldchange| > 1.4

**Table 6:** Functional enrichment analysis of differentially regulated^a ^genes elicited by TCDD in H4IIE cells using DAVID.

Category	Term	Gene count	Fold enrichment	P-value
**Enrichment Score: 3.05**				
GOTERM_BP_3	GO:0006805 ~ xenobiotic metabolic process	7	34.21	3.52E-08
GOTERM_BP_3	GO:0009410 ~ response to xenobiotic stimulus	7	31.47	6.09E-08
GOTERM_BP_3	GO:0006725 ~ cellular aromatic compound metabolic process	6	5.07	6.28E-03
GOTERM_BP_3	GO:0017144 ~ drug metabolic process	3	16.86	1.31E-02
GOTERM_BP_3	GO:0051186 ~ cofactor metabolic process	6	3.61	2.44E-02
GOTERM_BP_3	GO:0046483 ~ heterocycle metabolic process	5	1.78	3.03E-01
GOTERM_BP_3	GO:0044248 ~ cellular catabolic process	8	1.39	3.41E-01
				
**Enrichment Score: 2.45**				
GOTERM_BP_3	GO:0010035 ~ response to inorganic substance	11	4.70	1.00E-04
GOTERM_BP_3	GO:0006800 ~ oxygen and reactive oxygen species metabolic process	5	10.81	1.10E-03
GOTERM_BP_3	GO:0006979 ~ response to oxidative stress	7	4.50	4.38E-03
GOTERM_BP_3	GO:0044248 ~ cellular catabolic process	8	1.39	3.41E-01
				
**Enrichment Score: 1.59**				
GOTERM_BP_3	GO:0006629 ~ lipid metabolic process	14	2.49	3.37E-03
GOTERM_BP_3	GO:0006082 ~ organic acid metabolic process	12	2.71	4.12E-03
GOTERM_BP_3	GO:0042180 ~ cellular ketone metabolic process	12	2.68	4.57E-03
GOTERM_BP_3	GO:0044255 ~ cellular lipid metabolic process	11	2.77	5.68E-03
GOTERM_BP_3	GO:0005996 ~ monosaccharide metabolic process	7	4.23	5.88E-03
GOTERM_BP_3	GO:0016051 ~ carbohydrate biosynthetic process	5	6.18	8.38E-03
GOTERM_BP_3	GO:0044262 ~ cellular carbohydrate metabolic process	7	2.63	4.80E-02
GOTERM_BP_3	GO:0046165 ~ alcohol biosynthetic process	3	8.03	5.25E-02
GOTERM_BP_3	GO:0005975 ~ carbohydrate metabolic process	8	2.25	6.22E-02
GOTERM_BP_3	GO:0006091 ~ generation of precursor metabolites and energy	5	2.70	1.11E-01
GOTERM_BP_3	GO:0008610 ~ lipid biosynthetic process	4	1.71	4.09E-01
GOTERM_BP_3	GO:0044249 ~ cellular biosynthetic process	16	1.04	5.74E-01
				
**Enrichment Score: 1.58**				
GOTERM_BP_3	GO:0042493 ~ response to drug	11	3.94	4.20E-04
GOTERM_BP_3	GO:0006869 ~ lipid transport	4	3.91	8.07E-02
GOTERM_BP_3	GO:0010876 ~ lipid localization	4	3.54	1.01E-01
GOTERM_BP_3	GO:0042592 ~ homeostatic process	10	1.66	1.39E-01
				
**Enrichment Score: 1.54**				
GOTERM_BP_3	GO:0044255 ~ cellular lipid metabolic process	11	2.77	5.68E-03
GOTERM_BP_3	GO:0006721 ~ terpenoid metabolic process	3	10.54	3.20E-02
GOTERM_BP_3	GO:0006766 ~ vitamin metabolic process	3	4.75	1.29E-01

*^a^*Differentially regulated genes with P1(t) > 0.999 and |foldchange| > 1.4

Despite the nature of these continuous cell lines and the dysregulation of genes related to cell cycle control and regulation, GO terms associated with these functions were not enriched across all three cell lines. TCDD-treated Human HepG2 and mouse Hepa1c1c7 cell lines identified Functional clusters associated with cell cycle control (GO:0022402) were in enriched TCDD-treated HepG2 and Hepa1c1c7 gene expression data sets, but not in the H4IIE data set. Species-differences in functional clustering of TCDD-elicited differentially expressed gene data sets further corroborate species-specific responses to TCDD.

### Dioxin Response Element Analysis

Putative functional DREs are not equally distributed between species with more DREs associated with known human genes [17,18]. Within the human genome, there are 213,355 DRE cores in the proximal promoter regions of known genes (10kb upstream to 1kb downstream of a transcriptional start site [TSS]), with 139,289 and 100,614 DRE cores, in the mouse and rat genomes, respectively. To further investigate the divergent expression of CCND1, ID3 and GSTA5, their proximal promoter regions were searched for DRE cores. Human orthologs of CCND1 and ID3 contained 12 and 14 DRE cores, respectively, greater than the number found in either the mouse or rat genomes. In contrast, mouse and rat orthologs of GSTA5 each had 7 and 12 DRE cores, respectively, while the human only had 3 (Figures 7B and 7C). In each of these divergently regulated orthologs, the species with the greatest number of DRE cores within the regulatory region had the highest fold induction suggesting that species-specific regulons have important roles in regulating gene expression.

Each DRE core in the GSTA5 orthologs was extended by 7 bp on either end and assessed for sequence similarity by measuring the Euclidean distance between sequence pairs. Only the mouse and rat GSTA5 contained highly similar DRE sequences (Euclidian distance of ≤ 3.0; shaded rows in Figure 7C), and no human DRE sequences with high sequence similarity. Furthermore, the position of the conserved DRE sequence 5 bp upstream of the TSS in the mouse appears to be positionally conserved with the DRE sequence in rat the ortholog located 22 bp upstream of the TSS. Collectively, the disproportionate number of DREs between species and lack of sequence and spatially conserved DREs may account for the divergent regulation of human, mouse and rat GSTA5 orthologs.

## Discussion

This study comprehensively and systematically compares the gene expression responses elicited by TCDD across human, mouse and rat cells. Incorporating both custom cDNA microarrays to profile the temporal responses and more comprehensive commercial oligonucleotide microarrays, a limited number of conserved responses between species were identified. In addition, divergent and a large number of species-specific responses were identified that may contribute to species-specific differences in sensitivity and toxicity. These results are consistent with the poor response correlations of orthologous genes between C57BL/6 mice, Sprague Dawley and Long-Evans rats [10,11]. Collectively, reported *in vivo *rodent comparisons, and the *in vitro *data presented in this study suggest there are significant differences in TCDD elicited gene expression between species, despite the conservation of the AhR [4] and its signaling pathway.

*In vivo *and *in vitro *studies examining TCDD-elicited global gene expression have demonstrated that AhR targets a limited portion of the genome [10-12,19-23]. In addition, PWM-based computational searches identified a low percentage of orthologs with conserved putative functional DREs within their regulatory regions (10kb upstream of the TSS and the 5'UTR) [17,18]. Our comparative *in vitro *microarray results corroborate these findings. Temporal analysis using custom cDNA microarrays found that TCDD elicited a response in 9.9% of the represented genes in the HepG2 cells, 5.2% in the Hepa1c1c7 cells and only 1.1% in the H4IIE cells. Similar results were obtained using whole-genome microarrays where 4.7%, 2.4% and 0.7% of the genes exhibiting differential expression in HepG2, Hepa1c1c7 and H4IIE cells, respectively, at 24 hrs.

All three cell lines differentially expressed a core set of conserved gene responses that included the induction of CYP1A1, NQO1 and UGT1A6, members of the AhR gene battery [24]. However, a significant number of responses were specific to a single species (Figures 3B and 3B), as reported in *in vivo *studies [10,11]. Moreover, these studies not only identified species-specific responses, but also orthologs with divergent responses (i.e. same gene up-regulated in one species and down-regulated in other). Comparisons of C57BL/6 mouse and Sprague Dawley rat responses found that 29% of the commonly regulated orthologs exhibited divergent regulation [11]. Similarly, GSTA5, CCND1 and ID3, exhibited divergent regulation across HepG2, Hepa1c1c7 and H4IIE cells (Table 3). Each of these genes exhibited the same pattern with Hepa1c1c7 and H4IIE cells having comparable profiles while HepG2 cells exhibited the divergent response. For example, GSTA5 was up-regulated in Hepa1c1c7 and H4IIE cells, but down-regulated in HepG2 cells (Table 3). *In vivo *gene expression responses of GSTA5 orthologs matched the *in vitro *responses in the Hepa1c1c7 and H4IIE cells; TCDD treated mice and rats exhibited 1.8- and 1.9-fold maximum induction, respectively [11,12]. QRTPCR verified the divergent response of GSTA5 in the H4IIE and HepG2 cells, but did not detect a significant induction in the Hepa1c1c7 cells (Figure 7A).

Computational analysis of GST5A found a disproportionate number of DRE cores within the regulatory region sequence of each species (Figure 7B and 7C). Sequence analysis also found DRE sequences with high similarity in the mouse and rat orthologs, but not in the human GSTA5. The identification of species-specific DREs is consistent with the divergent regulation of GSTA5 between these cell lines. Overall, differences in ortholog expression may contribute to differences in TCDD sensitivity and toxicity across species.

Although these continuous cells lines are similar in morphology and were derived from hepatomas, there are inherent differences that may bias the identification of species-conserved and -divergent responses. For example, HepG2 cells were originally derived from the liver biopsy of a 15-year old Caucasian male and therefore may not be representative of a mature adult liver [25]. Recent studies have compared basal gene expression of liver samples to primary human hepatocytes, HepG2 and HepaRG cells, another human hepatoma cell line. Although, HepaRG cells were most similar to primary hepatocytes and liver samples [26], toxicogenomic studies report that HepaRG and HepG2 gene expression responses retained common functional processes [27]. In addition, the HepaRG donor differed in age and sex compared to the HepG2 patient, and was also infected with hepatitis C, which may affect both basal and TCDD-elicited gene expression. HepG2 cells were more sensitive in terms of the magnitude of regulation, and also in the terms of the total number of differentially regulated genes, and may be a more sensitive model for assessing TCDD exposure [27].

## Conclusion

Although a core set of conserved gene responses was identified, consistent with the role of AhR in mediating the adaptive metabolic responses, further evidence of differences in genome-wide gene expression profiles between species (i.e. species-specific regulons) is also presented. This is consistent with species-specific differences in TCDD sensitivity and toxicity [6,8,28,29], which are due to alterations in gene expression. Furthermore, there is a lack of conserved putative DREs within orthologous genes [18], and differences in genome-wide gene expression profiles has been reported between mice and rats *in vivo *[10,11]. Undoubtedly, the number of conserved responses and immaturity of the genome annotation, especially for the rat, limits the overall interspecies comparison [30]. Differences in AhR levels, co-activator availability, and protocols used in their isolation of these hepatoma cells may confound our comparisons. Nevertheless, the identification of numerous species-specific responses, evidence of divergent gene expression responses between species, and the discovery of distinct putative primary response sets in each cell line provides further compelling evidence that the effects of TCDD post-AhR binding are not conserved between species.

## Methods

### Cell Culture and Treatment

HepG2 (Dr. Trevor Archer, NIEHS, Research Triangle Park, NC), Hepa1c1c7 (Dr. Oliver Hankinson, University of California, Los Angeles, CA), and H4IIE (Dr. Niels Bols, University of Waterloo, Waterloo, Canada) cells were cultured in monolayers and treated with TCDD (S. Safe, Texas A&M University, College Station, TX) as previously described [16]. Briefly, cells were treated with either 10 nM TCDD or DMSO vehicle control for 1, 2, 4, 8, 12, 24 or 48 hrs for the time course studies. For co-treatment studies, cells were pretreated with 10 mg/ml cycloheximide (CHX; Sigma) for 1 hr and then treated for an additional 4 or 12 hrs with 10 nM TCDD or DMSO vehicle (Additional file 19). All treatment studies were performed in triplicate.

### RNA Isolation

Cells were harvested with 2.0 mL of TRIzol® Reagent (Invitrogen) and total RNA isolated according to the manufacturer's protocol followed by an acid phenol:chloroform extraction. Isolated RNA was resuspended in The RNA Storage Solution (Ambion Inc., Austin, TX), quantified (A_260_), and assessed for purity by measuring the A_260_/A_280 _ratio and by visual inspection of 1.0 μg on a denaturing gel.

### Microarray Experimental Design and Analysis

Gene expression changes were analyzed using custom human, mouse and rat cDNA microarrays as previously described [16,31,32]. Responses to CHX and TCDD co-treatment were also assayed with cDNA microarrays using a 2 × 2 factorial design (Additional file 20) [33]. Three replicates were performed with two independent labelings per sample (dye swap). In total, 42 cDNA microarrays were performed for each individual cell line. Additionally, 4 × 44 k Agilent Technologies whole-genome oligonucleotide microarrays (Santa Clara, CA), were used to profile the responses elicited by TCDD 24 hrs post-treatment according to the manufacturer's Two-Color Microarray-Based Gene Expression Analysis protocol Version 5.0.1, including dye swap labelings. Three replicates were performed for a total of 9 whole genome microarrays for each cell line. Microarray data quality was first assessed using a quality assurance protocol to ensure consistent high quality data throughout all studies prior to normalization and further analysis [34]. Microarray data were normalized using a semiparametric method [35], and statistically analyzed using an empirical Bayes methods [36]. The data were hierarchically clustered using the Euclidian distance in Cluster 3.0 [37] and visualized with Java Treeview [38]. Functional annotation clustering of Gene Ontology (GO) terms for differentially expressed genes was performed using DAVID (Database for Annotation, Visualization, and Integrated Discovery) [39]. Annotation clusters with an enrichment score ≥ 1.3 were considered significantly enriched. Networks of direct and indirect molecular interactions based on the whole-genome expression data were identified and visualized using the Ingenuity Pathway Analysis http://www.ingenuity.com.

### Quantitative Real-Time PCR

The same total RNA samples isolated for microarray studies were used for QRTPCR as previously described [16]. The copy number of each unknown sample was standardized to the geometric mean of three house-keeping genes (β-actin, Gapd, Hprt or Rpl13). Official gene names and symbols, RefSeq and Entrez Gene IDs, forward and reverse primer sequences, and amplicon sizes are provided in Additional file 21. Data were analyzed by analysis of variance (ANOVA) followed by Tukey's *post hoc *test using SAS 9.1 (SAS Institute, Cary, NC). Differences between treatment groups were considered significant when *p *< 0.05.

### Computational DREs Searches

The proximal promoter region (10 kb upstream and 1 kb downstream of a TSS) for a RefSeq corresponding to an individual gene were computationally searched for the substitute intolerant 5'-GCGTG-3' DRE core sequence and the position relative to the TSS were determined using the center of the 5 bp core (underlined). Each core was then extended by 7 bp upstream and downstream of the core to generate a 19 bp DRE core containing sequence, which were assigned a matrix similarity using a previously defined algorithm [18] and with an updated position weight matrix [17]. The 19 bp DRE core sequences from orthologous human, mouse and rat genes were hierarchically clustered in R http://www.R-project.org by measuring the Euclidean distance between pairs of sequences. DRE sequences that clustered together with a distance value ≤ 3.0 were characterized as orthologous DREs.

## Authors' contributions

ED performed the cell culture experiments, microarrays, QRTPCR cross-species comparison of the microarray data, computational DRE promoter analysis and the initial preparation of the manuscript. Additional QRTPCR was performed AWL. LDB normalized the microarray data and provided input into the microarray study designs. TRZ oversaw the completion of the study. All the authors have given final approval of the version to be published.

## Supplementary Material

Additional File 1 **HepG2 TCDD time course and cycloheximide cotreatment cDNA microarray data**. A table containing the expression ratios relative to the time matched vehicle control for the time course study. Ratios for the cycloheximide studies are relative to the treatment condition. P1(t)-values represent posterior probabilities of activity on a per gene and time-point basis or treatment condition using the model-based t-value. Click here for file

Additional File 2 **Hepa1c1c7 TCDD time course and cycloheximide cotreatment cDNA microarray data**. A table containing the expression ratios relative to the time matched vehicle control for the time course study. Ratios for the cycloheximide studies are relative to the treatment condition. P1(t)-values represent posterior probabilities of activity on a per gene and time-point basis or treatment condition using the model-based t-value. Click here for file

Additional File 3 **H4IIE TCDD time course and cycloheximide cotreatment cDNA microarray data**. A table containing the expression ratios relative to the time matched vehicle control for the time course study. Ratios for the cycloheximide studies are relative to the treatment condition. P1(t)-values represent posterior probabilities of activity on a per gene and time-point basis or treatment condition using the model-based t-value. Click here for file

Additional File 4 **Common differentially regulated orthologs elicited by TCDD in human HepG2 and mouse Hepa1c1c7 cells identified from the cDNA microarray time course studies**. A table containing the expression ratios for significantly differentially regulated orthologs (|fold change| > 1.4 and P1(t) > 0.999). Click here for file

Additional File 5 **Common differentially regulated orthologs elicited by TCDD in rat H4IIE and mouse Hepa1c1c7 cells identified from the cDNA microarray time course studies**. A table containing the expression ratios for significantly differentially regulated orthologs (|fold change| > 1.4 and P1(t) > 0.999). Click here for file

Additional File 6 **Common differentially regulated orthologs elicited by TCDD in human HepG2 and rat H4IIE cells identified from the cDNA microarray time course studies**. A table containing the expression ratios for significantly differentially regulated orthologs (|fold change| > 1.4 and P1(t) > 0.999). Click here for file

Additional File 7 **Putative human primary response genes and the number of DREs in their promoters**. A table listing the putative primary response genes identified from the cycloheximide studies and the number of 5'-GCGTG-3' DRE cores in the region 10 kb upstream and 1 kb downstream of a TSS. Click here for file

Additional File 8 **Putative mouse primary response genes and the number of DREs in their promoters**. A table listing the putative primary response genes identified from the cycloheximide studies and the number of 5'-GCGTG-3' DRE cores in the region 10 kb upstream and 1 kb downstream of a TSS. Click here for file

Additional File 9 **Putative rat primary response genes and the number of DREs in their promoters**. A table listing the putative primary response genes identified from the cycloheximide studies and the number of 5'-GCGTG-3' DRE cores in the region 10 kb upstream and 1 kb downstream of a TSS. Click here for file

Additional File 10 **Putative human secondary response genes and the number of DREs in their promoters**. A table listing the putative secondary response genes identified from the cycloheximide studies and the number of 5'-GCGTG-3' DRE cores in the region 10 kb upstream and 1 kb downstream of a TSS. Click here for file

Additional File 11 **Putative mouse secondary response genes and the number of DREs in their promoters**. A table listing the putative secondary response genes identified from the cycloheximide studies and the number of 5'-GCGTG-3' DRE cores in the region 10 kb upstream and 1 kb downstream of a TSS. Click here for file

Additional File 12 **Putative rat secondary response genes and the number of DREs in their promoters**. A table listing the putative secondary response genes identified from the cycloheximide studies and the number of 5'-GCGTG-3' DRE cores in the region 10 kb upstream and 1 kb downstream of a TSS. Click here for file

Additional File 13 **Whole-genome Agilent microarray data from HepG2 cells treated with TCDD for 24 hrs**. A table containing the expression ratio relative to the time matched vehicle control. P1(t)-values represent posterior probabilities of activity on a per gene basis using the model-based t-value. Click here for file

Additional File 14 **Whole-genome Agilent microarray data from Hepa1c1c7 cells treated with TCDD for 24 hrs**. A table containing the expression ratio relative to the time matched vehicle control. P1(t)-values represent posterior probabilities of activity on a per gene basis using the model-based t-value. Click here for file

Additional File 15 **Whole-genome Agilent microarray data from H4IIE cells treated with TCDD for 24 hrs**. A table containing the expression ratio relative to the time matched vehicle control. P1(t)-values represent posterior probabilities of activity on a per gene basis using the model-based t-value. Click here for file

Additional File 16 **Common differentially regulated orthologs elicited by TCDD in human HepG2 and mouse Hepa1c1c7 cells identified from the whole-genome Agilent microarrays at 24 hrs**. A table containing the expression ratios for significantly differentially regulated orthologs (|fold change| > 1.4 and P1(t) > 0.999). Click here for file

Additional File 17 **Common differentially regulated orthologs elicited by TCDD in rat H4IIE and mouse Hepa1c1c7 cells identified from the whole-genome Agilent microarrays at 24 hrs**. A table containing the expression ratios for significantly differentially regulated orthologs (|fold change| > 1.4 and P1(t) > 0.999). Click here for file

Additional File 18 **Common differentially regulated orthologs elicited by TCDD in human HepG2 and rat H4IIE cells identified from the whole-genome Agilent microarrays at 24 hrs**. A table containing the expression ratios for significantly differentially regulated orthologs (|fold change| > 1.4 and P1(t) > 0.999). Click here for file

Additional File 19 **Cell culture TCDD treatment and harvesting regimen**. For the time course studies, cells were treated with 10 nM TCDD or 0.1% DMSO vehicle and harvested at 1, 2, 4, 8, 12, 24, or 48 hrs post-treatment. For the cycloheximide studies cells were treated with 10 mg/ml cycloheximide 1 hr and then treated with either 10 nM TCDD or 0.1% DMSO vehicle and for an additional 4 or 12 hrs (as indicated by *). Click here for file

Additional File 20 **2 × 2 Factorial microarray experimental design used for the cycloheximide expression studies**. The hybridization design used to identify putative primary and secondary gene expression responses elicited by TCDD. Each arrow represents one microarray where the arrow heads and tails refer to Cy5 and Cy3 dye labeling, respectively. Double-headed arrows indicate dye swaps (each sample labeled with Cy3 and Cy5 on different microarrays). Click here for file

Additional File 21 **Gene symbols and primer sequences for QRTPCR**. A list of genes and primer sequences used to verify cDNA and Agilent microarray responses. Click here for file

## References

[B1] Poland A Knutson JC 2,3,7,8-tetrachlorodibenzo-p-dioxin and related halogenated aromatic hydrocarbons: examination of the mechanism of toxicity Annu Rev Pharmacol Toxicol 1982 22 517 554 10.1146/annurev.pa.22.040182.002505 6282188

[B2] Denison MS Heath-Pagliuso S The Ah receptor: a regulator of the biochemical and toxicological actions of structurally diverse chemicals Bulletin of environmental contamination and toxicology 1998 61 557 568 10.1007/PL00002973 9841714

[B3] Hankinson O The aryl hydrocarbon receptor complex Annu Rev Pharmacol Toxicol 1995 35 307 340 10.1146/annurev.pa.35.040195.001515 7598497

[B4] Schmidt JV Bradfield CA Ah receptor signaling pathways Annu Rev Cell Dev Biol 1996 12 55 89 10.1146/annurev.cellbio.12.1.55 8970722

[B5] Olson H Betton G Robinson D Thomas K Monro A Kolaja G Lilly P Sanders J Sipes G Bracken W Concordance of the toxicity of pharmaceuticals in humans and in animals Regul Toxicol Pharmacol 2000 32 56 67 10.1006/rtph.2000.1399 11029269

[B6] Schwetz BA Norris JM Sparschu GL Rowe UK Gehring PJ Emerson JL Gerbig CG Toxicology of chlorinated dibenzo-p-dioxins Environ Health Perspect 1973 5 87 99 10.2307/3428117 4270944 PMC1474972

[B7] Denison MS Vella LM Okey AB Structure and function of the Ah receptor for 2,3,7,8-tetrachlorodibenzo-p-dioxin. Species difference in molecular properties of the receptors from mouse and rat hepatic cytosols J Biol Chem 1986 261 3987 3995 3005314

[B8] Olson JR Holscher MA Neal RA Toxicity of 2,3,7,8-tetrachlorodibenzo-p-dioxin in the golden Syrian hamster Toxicol Appl Pharmacol 1980 55 67 78 10.1016/0041-008X(80)90221-5 7423509

[B9] Poland A Glover E Kende AS Stereospecific, high affinity binding of 2,3,7,8-tetrachlorodibenzo-p-dioxin by hepatic cytosol. Evidence that the binding species is receptor for induction of aryl hydrocarbon hydroxylase J Biol Chem 1976 251 4936 4946 956169

[B10] Boutros PC Yan R Moffat ID Pohjanvirta R Okey AB Transcriptomic responses to 2,3,7,8-tetrachlorodibenzo-p-dioxin (TCDD) in liver: comparison of rat and mouse BMC Genomics 2008 9 419 10.1186/1471-2164-9-419 18796159 PMC2559853

[B11] Boverhof DR Burgoon LD Tashiro C Sharratt B Chittim B Harkema JR Mendrick DL Zacharewski TR Comparative toxicogenomic analysis of the hepatotoxic effects of TCDD in Sprague Dawley rats and C57BL/6 mice Toxicol Sci 2006 94 398 416 10.1093/toxsci/kfl100 16960034

[B12] Fletcher N Wahlström D Lundberg R Nilsson CB Nilsson KC Stockling K Hellmold H Håkansson H 2,3,7,8-Tetrachlorodibenzo-p-dioxin (TCDD) alters the mRNA expression of critical genes associated with cholesterol metabolism, bile acid biosynthesis, and bile transport in rat liver: a microarray study Toxicol Appl Pharmacol 2005 207 1 24 10.1016/j.taap.2004.12.003 16054898

[B13] Lusska A Wu L Whitlock JP Superinduction of CYP1A1 transcription by cycloheximide. Role of the DNA binding site for the liganded Ah receptor J Biol Chem 1992 267 15146 15151 1321828

[B14] Ma Q Induction and superinduction of 2,3,7,8-tetrachlorodibenzo-rho-dioxin-inducible poly(ADP-ribose) polymerase: role of the aryl hydrocarbon receptor/aryl hydrocarbon receptor nuclear translocator transcription activation domains and a labile transcription repressor Arch Biochem Biophys 2002 404 309 316 10.1016/S0003-9861(02)00339-9 12147270

[B15] Joiakim A Mathieu PA Elliott AA Reiners JJ Superinduction of CYP1A1 in MCF10A cultures by cycloheximide, anisomycin, and puromycin: a process independent of effects on protein translation and unrelated to suppression of aryl hydrocarbon receptor proteolysis by the proteasome Molecular Pharmacology 2004 66 936 947 15385644 10.1124/mol.66.4.

[B16] Dere E Boverhof DR Burgoon LD Zacharewski TR In vivo-in vitro toxicogenomic comparison of TCDD-elicited gene expression in Hepa1c1c7 mouse hepatoma cells and C57BL/6 hepatic tissue BMC Genomics 2006 7 80 10.1186/1471-2164-7-80 16611356 PMC1513214

[B17] Dere E Forgacs AL Zacharewski TR Burgoon LD Genome-Wide Computational Analysis of Dioxin Response Element Location and Distribution in the Human, Mouse, and Rat Genomes Chem Res Toxicol 2011 21370876 10.1021/tx100328r PMC4038167

[B18] Sun YV Boverhof DR Burgoon LD Fielden MR Zacharewski TR Comparative analysis of dioxin response elements in human, mouse and rat genomic sequences Nucleic Acids Res 2004 32 4512 4523 10.1093/nar/gkh782 15328365 PMC516056

[B19] Jin B Kim G Park DW Ryu D-Y Microarray analysis of gene regulation in the Hepa1c1c7 cell line following exposure to the DNA methylation inhibitor 5-aza-2'-deoxycytidine and 2,3,7,8-tetrachlorodibenzo-p-dioxin Toxicol In Vitro 2004 18 659 664 10.1016/j.tiv.2004.02.006 15251184

[B20] McHale CM Zhang L Hubbard AE Zhao X Baccarelli A Pesatori AC Smith MT Landi MT Microarray analysis of gene expression in peripheral blood mononuclear cells from dioxin-exposed human subjects Toxicology 2007 229 101 113 10.1016/j.tox.2006.10.004 17101203

[B21] Puga A Maier A Medvedovic M The transcriptional signature of dioxin in human hepatoma HepG2 cells Biochem Pharmacol 2000 60 1129 1142 10.1016/S0006-2952(00)00403-2 11007951

[B22] Silkworth JB Carlson EA McCulloch C Illouz K Goodwin S Sutter TR Toxicogenomic analysis of gender, chemical, and dose effects in livers of TCDD- or aroclor 1254-exposed rats using a multifactor linear model Toxicol Sci 2008 102 291 309 10.1093/toxsci/kfm313 18178546

[B23] Xiong KM Peterson RE Heideman W Aryl hydrocarbon receptor-mediated down-regulation of sox9b causes jaw malformation in zebrafish embryos Molecular Pharmacology 2008 74 1544 1553 10.1124/mol.108.050435 18784347 PMC3147292

[B24] Nebert DW Roe AL Dieter MZ Solis WA Yang Y Dalton TP Role of the aromatic hydrocarbon receptor and [Ah] gene battery in the oxidative stress response, cell cycle control, and apoptosis Biochem Pharmacol 2000 59 65 85 10.1016/S0006-2952(99)00310-X 10605936

[B25] Ogino M Nagata K Yamazoe Y Selective suppressions of human CYP3A forms, CYP3A5 and CYP3A7, by troglitazone in HepG2 cells Drug Metab Pharmacokinet 2002 17 42 46 10.2133/dmpk.17.42 15618651

[B26] Hart SN Li Y Nakamoto K Subileau E-a Steen D Zhong X-b A comparison of whole genome gene expression profiles of HepaRG cells and HepG2 cells to primary human hepatocytes and human liver tissues Drug Metab Dispos 2010 38 988 994 10.1124/dmd.109.031831 20228232 PMC2879958

[B27] Jennen DGJ Magkoufopoulou C Ketelslegers HB van Herwijnen MHM Kleinjans JCS van Delft JHM Comparison of HepG2 and HepaRG by whole-genome gene expression analysis for the purpose of chemical hazard identification Toxicol Sci 2010 115 66 79 10.1093/toxsci/kfq026 20106945

[B28] Bickel M Polychlorinated persistent compounds Experientia 1982 38 879 882 10.1007/BF01953634 6813132

[B29] Vos JG Moore JA Zinkl JG Toxicity of 2,3,7,8-tetrachlorodibenzo-p-dioxin (TCDD) in C57B1/6 mice Toxicol Appl Pharmacol 1974 29 229 241 10.1016/0041-008X(74)90060-X 4283688

[B30] Boverhof DR Zacharewski TR Toxicogenomics in risk assessment: applications and needs Toxicol Sci 2006 89 352 360 10.1093/toxsci/kfj018 16221963

[B31] Kim S Dere E Burgoon LD Chang C-C Zacharewski TR Comparative analysis of AhR-mediated TCDD-elicited gene expression in human liver adult stem cells Toxicol Sci 2009 112 229 244 10.1093/toxsci/kfp189 19684285 PMC2769060

[B32] Kwekel JC Forgacs AL Burgoon LD Williams KJ Zacharewski TR Tamoxifen-elicited uterotrophy: cross-species and cross-ligand analysis of the gene expression program BMC medical genomics 2009 2 19 10.1186/1755-8794-2-19 19400957 PMC2683873

[B33] Yang YH Speed T Design issues for cDNA microarray experiments Nat Rev Genet 2002 3 579 588 12154381 10.1038/nrg863

[B34] Burgoon L Eckel-Passow J Gennings C Boverhof D Burt J Fong C Zacharewski T Protocols for the assurance of microarray data quality and process control Nucleic Acids Res 2005 33 e172 10.1093/nar/gni167 16272462 PMC1278948

[B35] Eckel J Gennings C Therneau T Burgoon L Boverhof D Zacharewski T Normalization of two-channel microarray experiments: a semiparametric approach Bioinformatics 2005 21 1078 1083 10.1093/bioinformatics/bti105 15513988

[B36] Eckel J Gennings C Chinchilli V Burgoon L Zacharewski T Empirical bayes gene screening tool for time-course or dose-response microarray data J Biopharm Stat 2004 14 647 670 10.1081/BIP-200025656 15468757

[B37] de Hoon MJL Imoto S Nolan J Miyano S Open source clustering software Bioinformatics 2004 20 1453 1454 10.1093/bioinformatics/bth078 14871861

[B38] Saldanha AJ Java Treeview--extensible visualization of microarray data Bioinformatics 2004 20 3246 3248 10.1093/bioinformatics/bth349 15180930

[B39] Dennis G Sherman BT Hosack DA Yang J Gao W Lane HC Lempicki RA DAVID: Database for Annotation, Visualization, and Integrated Discovery Genome Biol 2003 4 P3 10.1186/gb-2003-4-5-p3 12734009 PMC193660

