# Supplementary figures and images for: Differences in TCDD-elicited gene expression profiles in human HepG2, mouse Hepa1c1c7 and rat H4IIE hepatoma cells
Source: BMC Genomics. 2011 Apr 15;12:193. doi: 10.1186/1471-2164-12-193 (PMC3089798; doi:10.1186/1471-2164-12-193)

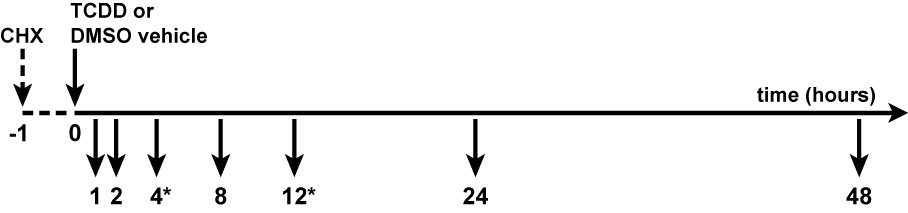

Supplement: Additional File 19 — Cell culture TCDD treatment and harvesting regimen. For the time course studies, cells were treated with 10 nM TCDD or 0.1% DMSO vehicle and harvested at 1, 2, 4, 8, 12, 24, or 48 hrs post-treatment. For the cycloheximide studies cells were treated with 10 mg/ml cycloheximide 1 hr and then treated with either 10 nM TCDD or 0.1% DMSO vehicle and for an additional 4 or 12 hrs (as indicated by *). [file 1471-2164-12-193-S19.JPEG]

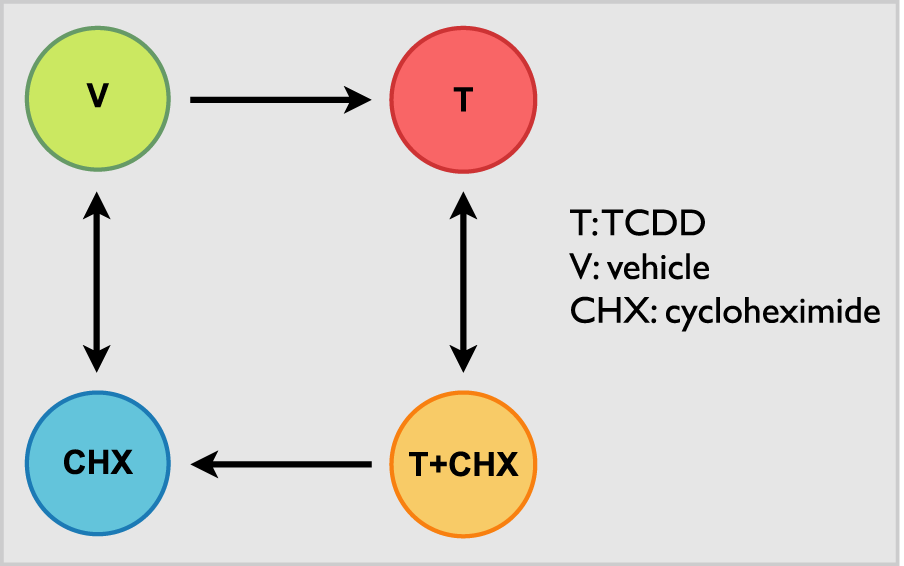

Supplement: Additional File 20 — 2 × 2 Factorial microarray experimental design used for the cycloheximide expression studies. The hybridization design used to identify putative primary and secondary gene expression responses elicited by TCDD. Each arrow represents one microarray where the arrow heads and tails refer to Cy5 and Cy3 dye labeling, respectively. Double-headed arrows indicate dye swaps (each sample labeled with Cy3 and Cy5 on different microarrays). [file 1471-2164-12-193-S20.PNG]
